# LncRNA‐ZXF1 stabilizes P21 expression in endometrioid endometrial carcinoma by inhibiting ubiquitination‐mediated degradation and regulating the miR‐378a‐3p/PCDHA3 axis

**DOI:** 10.1002/1878-0261.12940

**Published:** 2021-03-22

**Authors:** Deshui Kong, Yixin Hou, Wenzhi Li, Xiaohong Ma, Jie Jiang

**Affiliations:** ^1^ Department of Obstetrics and Gynecology Qilu Hospital of Shandong University Jinan China

**Keywords:** cell cycle, endometrioid endometrial cancer, lncRNA‐ZXF1, P21, ubiquitination

## Abstract

Long noncoding RNAs (lncRNAs) have a profound effect on biological processes in various malignancies. However, few studies have investigated their functions and specific mechanisms in endometrial cancer. In this study, we focused on the role and mechanism of lncRNA‐ZXF1 in endometrial cancer. Bioinformatics and *in vitro* and *in vivo* experiments were used to explore the expression and function of lncRNA‐ZXF1. We found that lncRNA‐ZXF1 altered the migration and invasion of endometrioid endometrial cancer (EEC) cells. Furthermore, our results suggest that lncRNA‐ZXF1 regulates EEC cell proliferation. This regulation may be achieved by the lncRNA‐ZXF1‐mediated alteration in the expression of P21 through two mechanisms. One is that lncRNA‐ZXF1 functions as a molecular sponge of miR‐378a‐3p to regulate PCDHA3 expression and then modulate the expression of P21. The other is that lncRNA‐ZXF1 inhibits CDC20‐mediated degradation of ubiquitination by directly binding to P21. To the best of our knowledge, this study is the first to explore lncRNA‐ZXF1 functioning as a tumor‐suppressing lncRNA in EEC. LncRNA‐ZXF1 may become therapeutic, diagnostic, and prognostic indicator in the future.

## Graphical abstract

In this study, we found that lncRNA‐ZXF1 may act as a tumor suppressor gene to regulate the proliferation and invasion of endometrioid endometrial cancer (EEC). LncRNA‐ZXF1 potentially alters P21 expression in EEC through two mechanisms: by regulating P21 protein expression through the miR‐378a‐3p/PCDHA3 axis and by directly binding to P21 to prevent its CDC20‐mediated ubiquitin degradation.

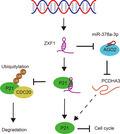

**Abbreviations**
3'UTR: 3'Untranslated region
CDC20: cell division cycle protein 20
ceRNA: competing endogenous RNA
DFS: disease‐free survival
EEC: endometrioid endometrial carcinoma
GSEA: Gene Set Enrichment Analysis
LncRNA: long noncoding RNA
OS: overall survival
P21: cyclin‐dependent kinase inhibitor 1A (P21)
PCDHA3: protocadherin alpha‐3
ROC: receiver operating characteristic curve
UCEC: uterine corpus endometrial carcinoma
ZXF1: ACTA2 antisense RNA 1 (ZXF1)

## 1 Introduction

Uterine corpus endometrial carcinoma (UCEC) is a malignant tumor with high morbidity and mortality rates worldwide [1, 2]. The ever‐increasing number of cases and incurable conditions have led scientists to identify new treatments and predictive indicators [3, 4]. The malignant characteristics of UCEC are mainly manifested by the uncontrolled self‐replication of cancer cells and aggressive metastasis to surrounding tissues [5]. The accelerated replication cycle of tumor cells provides a cytological basis for the immortalization and invasive metastasis of tumors [6, 7, 8]. The development of sensitive and specific targeted therapies for postoperative use is urgently needed.

Long noncoding RNAs (lncRNAs) are single‐stranded RNAs that are longer than 200 nt but do not encode proteins [9, 10]. An increasing number of studies have confirmed that lncRNAs play an important role in biological processes [9, 11]. Many studies have found that lncRNAs act as a competitive endogenous RNA(ceRNA) [12]. There are also lncRNAs that may affect protein post‐translational modifications, such as ubiquitination [13, 14]. Research on lncRNAs may provide a new understanding of malignant tumors and help us research and treat malignant tumors. LncRNA‐ZXF1 is a new lncRNA with a length of 2450 nt that is transcribed from chromosome 10. According to recent studies, lncRNA‐ZXF1 functions in cervical cancer [15], lung cancer [16, 17], and liver cancer [18]. However, these studies did not thoroughly study the mechanism of lncRNA‐ZXF1. To date, no study has investigated the function and mechanism of lncRNA‐ZXF1 in endometrioid endometrial cancer (EEC). This study fills the gap in research on the role of lncRNA‐ZXF1 in EEC.

In this study, we investigated the expression and function of lncRNA‐ZXF1 in EEC using bioinformatics and in vivo and in vitro experiments. LncRNA‐ZXF1 may regulate the migration, invasion, and proliferation of EEC cells. LncRNA‐ZXF1 potentially alters the expression of P21 through two mechanisms: controlling the expression of P21 through the ZXF1/miR‐378a‐3p/PCDHA3 axis and binding to P21 to prevent the degradation of P21 by the ubiquitin–proteasome system. Based on these findings, ZXF1 has the potential to become a diagnostic indicator, prognostic indicator, and target for molecular targeted treatment in precision therapy.

## 2 Materials and methods

### 2.1 Acquisition of clinical samples

Pathology‐confirmed endometrial carcinoma tissues were obtained from patients who had undergone surgery at the Gynecology Department of Shandong University Qilu Hospital. All the patients were informed about the experimental details and provided written informed consent. The experimental procedure complied with the Declaration of Helsinki and was authorized by the Ethics Committee on Scientific Research of Shandong University Qilu Hospital.

### 2.2 Cell culture

Ishikawa and HEC‐1A cell lines were purchased from ZhongQiaoXinZhou Biotechnology Co., Ltd. (Shanghai, China). Ishikawa cells were cultured in RPMI 1640 medium (BI, Israel), and HEC‐1A cells were cultured in McCoy’s 5A medium (Macgene, Beijing, China). Both media were supplemented with 10% fetal bovine serum (FBS), and cells were cultured at 4 °C in a humidified atmosphere containing 5% CO_2_. All cell lines were mycoplasma‐free, and short tandem repeats (STRs) were confirmed.

### 2.3 In situ hybridization of ZXF1

The Ribo™ Fluorescence In Situ Hybridization Kit (RiboBio, Guangzhou, China) was used to perform RNA fluorescence in situ hybridization. All the manipulations were performed according to the manufacturer’s protocols. Probes that specifically target ZXF1 were designed and synthesized by RiboBio.

### 2.4 Cell proliferation

Cell viability was determined using the colony formation assay, 3‐(4, 5‐dimethylthiazol‐2‐yl)‐2, 5‐diphenyltetrazolium bromide (MTT) assay (Sigma, St. Louis, MO, USA) and Celltiter‐Glo assay (Promega, Madison, WI, USA). The 5‐ethynyl‐20‐deoxyuridine (EdU) incorporation assay kit (RiboBio) was used to detect nuclear replication according to the manufacturer’s instructions. Propidium iodide (PI) was utilized to stain ethanol‐treated cells. Next, the cell cycle distribution was detected using a FACSCalibur flow cytometer (BD Biosciences, Franklin Lakes, NJ, USA).

### 2.5 Tumor cell migration assay and wound healing assay

Transwell assays were performed using Transwell chambers (Corning Life Sciences, Corning, NY, USA) coated with or without Matrigel (BD Biosciences). A total of 1.5 × 10^5^ cells was added to the upper chamber of the insert. As a chemoattractant, 700 µL of medium containing 20% FBS was added to the lower chamber. After an incubation for 18–36 h, the cells on the lower surface were fixed with ethanol and stained with 0.2% crystal violet. Images were captured using an Olympus IX51‐inverted microscope. The number of migrated or invaded cells was counted in five randomly selected fields (magnification, ×200) from each chamber under the microscope and the relative cell number was calculated.

A total of 2 × 10^5^ cells were seeded in each well of 24‐well plates, incubated overnight, and scratch wounds were generated using 10‐μL pipette tips. The cells were then incubated with serum‐free medium and photographed at the same position at 0, 24, 48, and 72 h. The area that had healed at each time point relative to 0 h was used to calculate the amount of migration.

### 2.6 Western blotting

Cells were washed three times with PBS and lysed on ice using radioimmunoprecipitation assay buffer (RIPA; Beyotime Institute of Biotechnology, Haimen, China) containing 1% phenylmethylsulfonyl fluoride (PMSF) and 1% NaF for 30 min. Cell lysates were centrifuged at 13 362 ***g*** for 10 min at 4 °C, and the protein extracts (30–50 µg) were loaded in each lane and separated by sodium dodecyl sulfate/polyacrylamide gel electrophoresis. Then, cellular proteins were transferred to polyvinylidene difluoride membranes (Millipore, Billerica, MA, USA) after extraction and electrophoretic separation. Next, the membranes were incubated with primary and secondary antibodies. The blots were visualized using the Immobilon^®^ Western horseradish peroxidase substrate (Millipore). The specific information on the primary antibodies used in this study is provided in Table S1.

### 2.7 Quantitative real‐time reverse transcription–PCR and plasmid extraction

Total RNA was purified using TRIzol reagent (Invitrogen, Carlsbad, CA, USA). Reverse transcription was performed using the M‐MLV system (Cat no. C28025‐011; Invitrogen, China). qRT‐PCR quantified RNA expression using the SYBR Green Master Mix (Takara, Japan). ACTB and U6 were used as internal controls for mRNAs and miRNAs, respectively. The relative RNA abundance was calculated using the standard 2^‐ΔΔCt^ method. The primers used in the present study are listed in Table S2. Plasmid extraction was performed according to the manufacturer’s protocols (Omega Bio‐tek, Norcross, GA, USA).

### 2.8 Construction and transfection of plasmids or lentiviruses

The CDC20 cDNA was amplified, inserted into the pCMV‐myc vector (Beyotime, China), and then verified by sequencing. The miR‐378a‐3p inhibitor and mimics were purchased from GenePharma (Shanghai, China). Transfection was performed using Lipofectamine 2000 (Invitrogen) according to the manufacturer’s protocol. Lentiviruses overexpressing ZXF1 and PCDHA3 were purchased from GeneChem (Shanghai, China).

### 2.9 RNA immunoprecipitation (RIP)

RIP assays were conducted using the EZ‐Magna RIP RNA‐binding Protein Immunoprecipitation Kit (Millipore, Burlington, MA, USA) and standard protocols. The collected cells were lysed on ice with RIP lysis buffer and stored at −80 °C after lysis. Fifty microliters of the magnetic bead suspension was washed, 5 µg of target primary antibody was added, and the mixture was rotated and incubated for 30 min at room temperature. IgG was used as a negative control for protein immunoprecipitation during the experiment. After washing the magnetic beads, the thawed cell lysate was added and rotated overnight at 4 °C. Proteins were digested with proteinase K, and then, the RNA was purified and reverse‐transcribed into cDNAs for q‐PCR experiments.

### 2.10 Immunohistochemistry (IHC)

Tissues were embedded in paraffin and cut into 4‐µm paraffin sections. Tissue sections were dewaxed with xylene and rehydrated in a graded alcohol series, and then, antigen retrieval was performed in citric acid antigen repair buffer (pH = 6.0). After cooling to room temperature, tissue sections were incubated with 3% hydrogen peroxide and covered with BSA to block nonspecific binding. Next, the tissue sections were incubated with diluted primary antibodies against Ki67 and P21 at 4°C overnight. After an incubation with the corresponding secondary antibody, the sections were stained with DAB and then counterstained with hematoxylin. Quant Center software (from 3D HISTECH, Budapest, Hungary) was used to analyze the results. The specific information on the primary antibody used in the present study is provided in Table S1.

### 2.11 Immunoprecipitation

Cells were transiently transfected with PCDHA3‐Flag, CDC20‐Myc, and ZXF1 using standard experimental procedures. After the incubation, cells were lysed with NETN buffer (20 mm Tris/HCl [pH = 8.0], 100 mm NaCl, 1 mm EDTA, 0.5% NP‐40, and multiple protease inhibitors). The lysate was incubated with Protein A/G agarose beads (Dallas, TX, USA) and antibodies overnight. The beads were washed 5 times with NETN buffer, and all immunoprecipitated proteins were detected using western blotting.

### 2.12 Dual‐luciferase reporter assay

The wild‐type and mutant 3ʹ UTR of PCDHA3 were cloned into the GV272 vector (GeneChem, Shanghai, China). The dual‐luciferase reporter assay system (Promega) was used to explore luciferase activity.

### 2.13 Xenograft model and small live animal imaging technology

BALB/c nude mice (female, aged 6 weeks, 13.8 ± 1.9 g) were purchased from Beijing Vital River Laboratory Animal Technology Co., Ltd. (Beijing, China) and housed in SPF breeding units. Using the method of random allocation, the mice were equally allocated to two squirrel cages and treated differently. All mice were injected subcutaneously with 8 × 10^6^ tumor cells. Before the experimental mice were sacrificed, the IVIS Imaging System (PerkinElmer, Waltham, MA, USA) was used to examine the fluorescence intensity of the tumor. The tumor volume was calculated as follows: tumor volume = width^2^× length/2. The tumor mass was weighed after the mice were sacrificed. qRT‐PCR and IHC were used to measure the expression levels of target molecules. All animal experiments were reviewed and approved by the Laboratory Animal Ethics and Welfare Committee of Shandong University Cheeloo College of Medicine.

### 2.14 Statistical analysis and bioinformatics

All experiments were repeated at least 3 times independently. The endometrial cancer gene expression data and clinical phenotype information were downloaded from The Cancer Genome Atlas (TCGA). The GSE17025 data were downloaded from the GEO database. The data were analyzed and mapped using GraphPad Prism 8.0 software (San Diego, CA, USA). Statistical significance was determined using Student’s *t*‐test, the Mann–Whitney *U*‐test, and ANOVA. Correlation analyses and receiver operating characteristic (ROC) curve analyses were performed using SPSS 22.0 (Chicago, IL, USA). Differences at *P* < 0.05 were considered significant for all statistical tests. Molecular genomic information was downloaded from the UCSC site (http://www.genome.ucsc.edu/index.html). Information on the binding of miR‐378a‐3p to long‐stranded RNA was obtained from ENCORI (http://starbase.sysu.edu.cn/). GSEA software (Broad Institute, USA) was utilized to enrich pathways in TCGA data.

## 3 Results

### 3.1 Low expression of ZXF1 indicates a poor prognosis for patients with UCEC

**Fig. 1:**
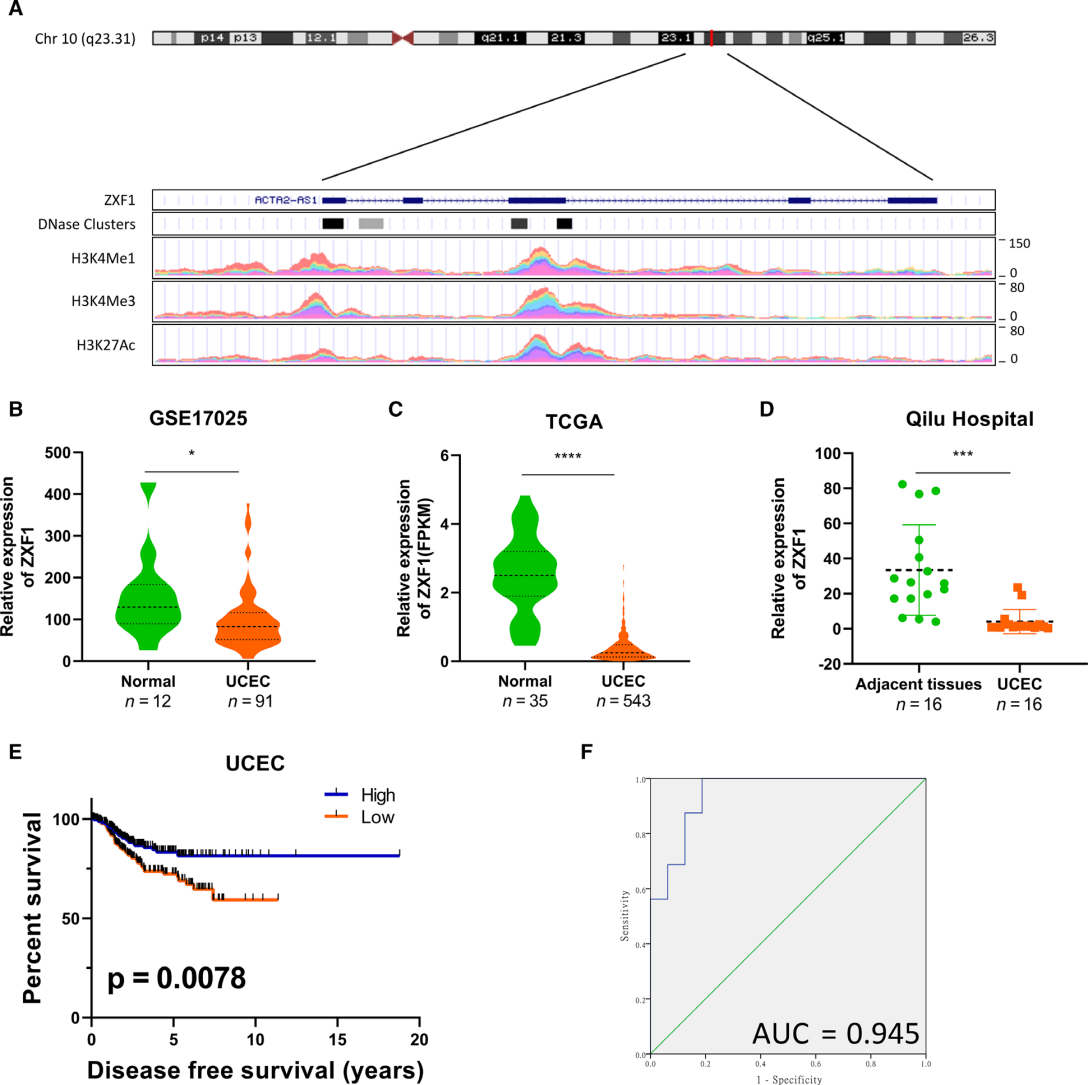
LncRNA‐ZXF1 has diagnostic and prognostic potential in UCEC. (A) ZXF1 is located on chromosome 10 (q23.31). ENCODE and UCEC data predict the transcriptional regulation of ZXF1 and indicate the ZXF1 promoter region. H3K27Ac mark (often found near regulatory elements) in 7 cell lines and DNase I hypersensitivity peak clusters from ENCODE (95 cell lines). (B and C). In the normalized GSE17025 (B) and TCGA (C) data, ZXF1 expression was reduced in tumor tissues compared with control samples. The *P*‐value was obtained from the Mann–Whitney *U*‐test. The violin plot displays the overall distribution of the data, including the medians and quartile numbers. (D) Sixteen pairs of endometrial cancer and adjacent tissues were collected. The qRT‐PCR results showed that ZXF1 expression was also lower in tumor samples. The *P*‐value was obtained from the Mann–Whitney *U*‐test. (E) A Kaplan–Meier analysis was performed to determine prognostic value of ZXF1. The median ZXF1 expression in TCGA data was used to divide the samples into two groups. Half of the patients with high ZXF1 expression had a longer disease‐free survival time. The *P*‐value was calculated using the log‐rank test. (F) The ROC curve was used to show the diagnostic value of ZXF1 in samples from Qilu Hospital.

LncRNA‐ZXF1, also called ACTA2‐AS1 and uc001kfo, is an antisense lncRNA located on chromosome 10 (q23.31). Both activated histone–protein modifications and DNase I‐hypersensitive sites frequently appear around the transcription promoter region of ZXF1 (ENCODE and UCSC data) (Fig. 1A). ZXF1 also showed differential expression in other malignant tumors in TCGA data (Fig. S1). The secondary structure of lncRNA‐ZXF1 was predicted by the RNAFold web server (http://rna.tbi.univie.ac.at/cgi‐bin/RNAWebSuite/RNAfold.cgi) (Fig. S2a). To understand the changes in the expression of ZXF1 in UCEC, FPKM data and the GSE17025 dataset were downloaded from TCGA and GEO databases, respectively. The expression of ZXF1 in UCEC was lower than normal tissues from the GSE17025 (Fig. 1B) and TCGA (Fig. 1C) data. An analysis of ZXF1 expression in TCGA data stratified according to stage showed that each stage of UCEC showed differential expression compared with normal tissues, but no difference in expression was observed between stages (Fig. S2b). Sixteen UCEC samples and adjacent tissues were collected from Shandong University Qilu Hospital, and the qRT‐PCR results showed the same trend (Fig. 1D). An analysis of the disease‐free survival (DFS) of patients in TCGA revealed that patients with UCEC presenting high levels of ZXF1 had a longer disease‐free survival (Fig. 1E). Although no significant difference in overall survival (OS) was identified using the log‐rank test, high expression may be beneficial to the long‐term survival prognosis (Fig. S2c). The receiver operating characteristic (ROC) curve analysis using data obtained from Qilu Hospital showed that ZXF1 has good specificity and sensitivity for distinguishing adjacent tissues from cancerous tissues (Fig. 1F). Similar results were also obtained from TCGA and GEO databases (Fig. S2.d). Thus, ZXF1 may have the potential to become an indicator of the clinical diagnosis and prognosis.

### 3.2 ZXF1 arrests the cell cycle in endometrial cancer

**Fig. 2:**
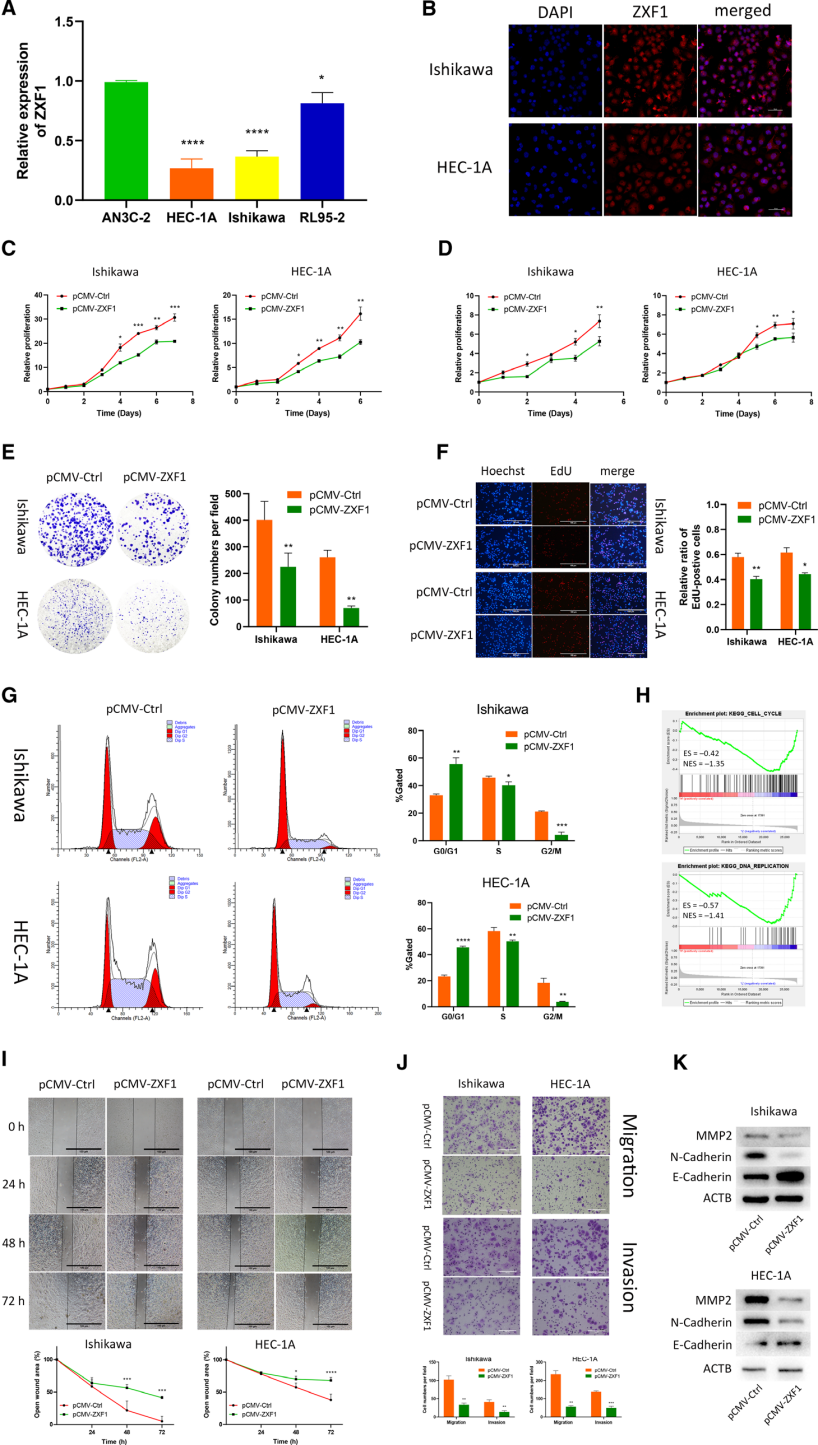
LncRNA‐ZXF1 overexpression reduces the viability and invasion of EEC cells. (A) Comparison of ZXF1 expression in the EEC cell lines using qRT‐PCR. (B) Fluorescence in situ hybridization of ZXF1 was performed in EEC cell lines. ZXF1 was detected in both the cytoplasm and nucleus. Scale bars, 50 μm. (C) The Celltiter‐Glo curves showed that ZXF1 reduced the viability of EECs. Comparisons among multiple groups are analyzed using two‐way ANOVA. (D) The MTT assay showed that ZXF1 inhibited the proliferation of endometrial tumor cells. Comparisons among multiple groups are analyzed using two‐way ANOVA. (E) Colony formation experiments proved that ZXF1 reduces the number of colonies formed from single EEC cells. (F) DNA replication in the nucleus was detected using the EdU incorporation assay. After ZXF1 overexpression, DNA replication was inhibited. Scale bars, 100 μm. (G) Flow cytometry showed that ZXF1 arrested the cell cycle in Ishikawa and HEC‐1A cells. (H) All samples were divided into two groups according to the expression of ZXF1 in TCGA, and then, a GSEA was performed to enrich the cell cycle and DNA replication results. (I) Overexpression of ZXF1 in tumor cells used in the wound healing experiment inhibited migration. Scale bars, 100 μm. (J) Transwell migration and invasion assays were performed using Ishikawa and HEC‐1A cells transfected with pCMV‐ZXF1. The presence of ZXF1 weakened the migration and invasion capabilities of tumor cells. Scale bars, 100 μm. (K) Protein expression levels of invasion‐related markers in Ishikawa and HEC‐1A cells transfected with ZXF1 and control vectors. All data are mean ± SD. Significance calculated using the unpaired t‐test. *P < 0.05, **P < 0.01, ***P < 0.001,****P < 0.0001. Representative data are from three independent experiments.

ZXF1 expression was detected in endometrial carcinoma cell lines using qRT‐PCR to identify a suitable cell line for in vitro experiments (Fig. 2A). ZXF1 was expressed at lower levels in Ishikawa and HEC‐1A cells than in two other cell lines. Because ZXF1 was downregulated in EECs, we chose Ishikawa and HEC‐1A cells for in vitro experiments. Fluorescence in situ hybridization (FISH) was performed to explore the subcellular localization of ZXF1 in endometrial cancer cells. FISH (Fig. 2B) showed that ZXF1 is located in the nucleus and cytoplasm simultaneously.

According to the results of ZXF1 expression in tumors and the survival analysis, we assumed that ZXF1 is a tumor suppressor gene. Celltiter‐Glo (Fig. 2C) and MTT (Fig. 2D) assays were performed to analyze the viability and proliferation of Ishikawa and HEC‐1A cells overexpressing ZXF1. ZXF1 inhibited the proliferation of EEC cells. In colony formation experiments, ZXF1 overexpression suppressed the colony formation ability of Ishikawa and HEC‐1A cells (Fig. 2E). The EdU assay was utilized to clarify the function of ZXF1 in DNA replication and revealed that high expression of ZXF1 obstructed active DNA replication in the nucleus of EEC cells (Fig. 2F). The proportion of cells in each phase of the cell cycle was measured using flow cytometry after propidium iodide (PI) staining. ZXF1 overexpression increased the proportion of cells in G0/G1 phase and reduced the proportion of cells in the S and G2/M phases (Fig. 2G). We performed a GSEA with TCGA data to explore the relationship between ZXF1 and the cell cycle. The median ZXF1 expression was used as the cutoff value, and all TCGA samples were divided into two groups. Cell cycle (ES = −0.42 and NES = −1.35) and DNA replication (ES = −0.57 and NES = −1.41) were enriched after calculation using KEGG pathways as the background (Fig. 2H). In the aforementioned experiments, we used various methods to confirm that ZXF1 could affect the cell cycle of EEC cells.

Wound healing and Transwell assays were performed to test the function of ZXF1 in cell migration and invasion. Transwell experiments (Fig. 2J) and scratch wounds observed at different time points (Fig. 2I) confirmed that ZXF1 inhibited tumor migration and invasion. We also measured the protein expression of markers of tumor cell invasion. Compared with the control group, MMP2, N‐cadherin, and E‐cadherin showed different expression levels in ZXF1‐transfected cells using western blotting (Fig. 2K). These data prove that ZXF1 may also play a role in tumor migration and invasion.

### 3.3 ZXF1 inhibits the growth of subcutaneous EEC tumors *in vivo*

**Fig. 3:**
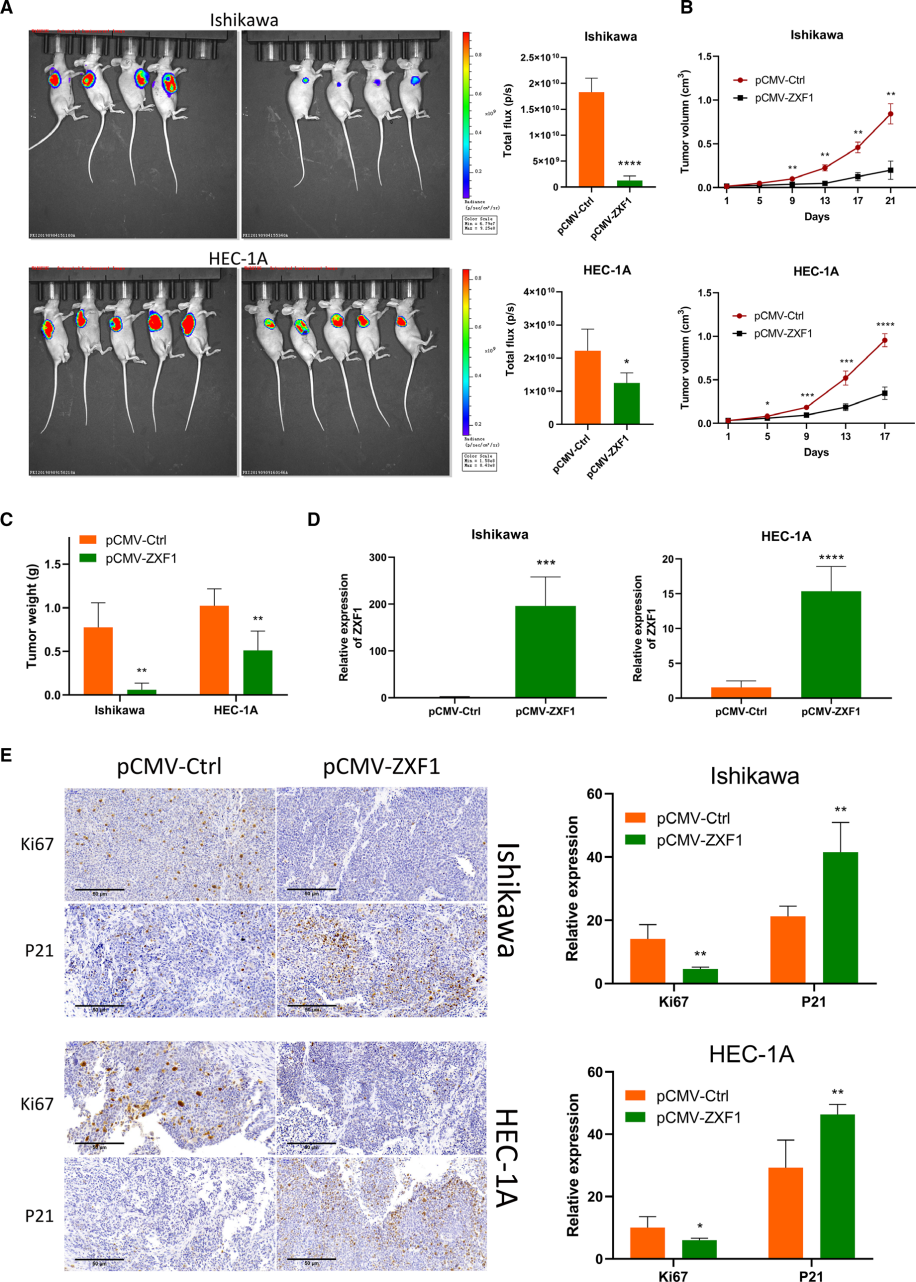
ZXF1 inhibits the growth of subcutaneous EEC tumors in vivo. (A) Subcutaneous inoculation of EEC cells expressing luciferase was performed in the armpits of mice to induce tumorigenesis. Total flux calculations showed the tumor suppressor function of ZXF1 in vivo. (B) Growth of subcutaneous tumors over time. Comparisons among multiple groups are analyzed using two‐way ANOVA. (C) After the mice were sacrificed, the comparison of tumor weights suggested that ZXF1 expression was not conducive to the growth of subcutaneous tumors. (D) Expression of ZXF1 in mouse tumor samples. e. Relative expression of the Ki67 and P21 proteins in mouse tumor samples. Scale bars, 50 μm. All data are mean ± SD. Significance calculated using the unpaired *t*‐test. *P < 0.05, **P < 0.01, ***P < 0.001,****P < 0.0001. Representative data are from three independent experiments.

In vivo experiments were designed and conducted to verify the reliability of the experimental results in vitro. Luciferase lentivirus‐infected EEC cells overexpressing ZXF1 and normal controls were evaluated. Two groups of 6‐week‐old female mice were injected subcutaneously with 8 × 10^6^ treated cells. Similar experiments were performed using the two EEC cell lines to make the results more robust. After the subcutaneous injection of the tumor cell suspension into nude mice, the tumor size and survival status were regularly observed. In vivo imaging of both cell lines revealed that ZXF1 reduced the tumor growth rate (Fig. 3A). The same conclusion was drawn from the tumor volume growth curve (Fig. 3B). After the humane euthanasia of experimental animals, the subcutaneous tumor masses were removed and weighed. The tumor mass of the experimental group overexpressing ZXF1 was smaller than the control group (Fig. 3C). A portion of the tumor mass was isolated for qRT‐PCR experiments to verify ZXF1 expression (Fig. 3D). Another portion of the tumor was embedded in paraffin and cut into sections for IHC. As a marker of proliferation, Ki67 and P21 antibodies were used in IHC experiments. The expression of Ki67 in the control group was much higher than in the ZXF1 group, and P21 expression showed the opposite trend (Fig. 3E).

### 3.4 ZXF1 regulates PCDHA3 expression as a sponge of miR‐378a‐3p

**Fig. 4:**
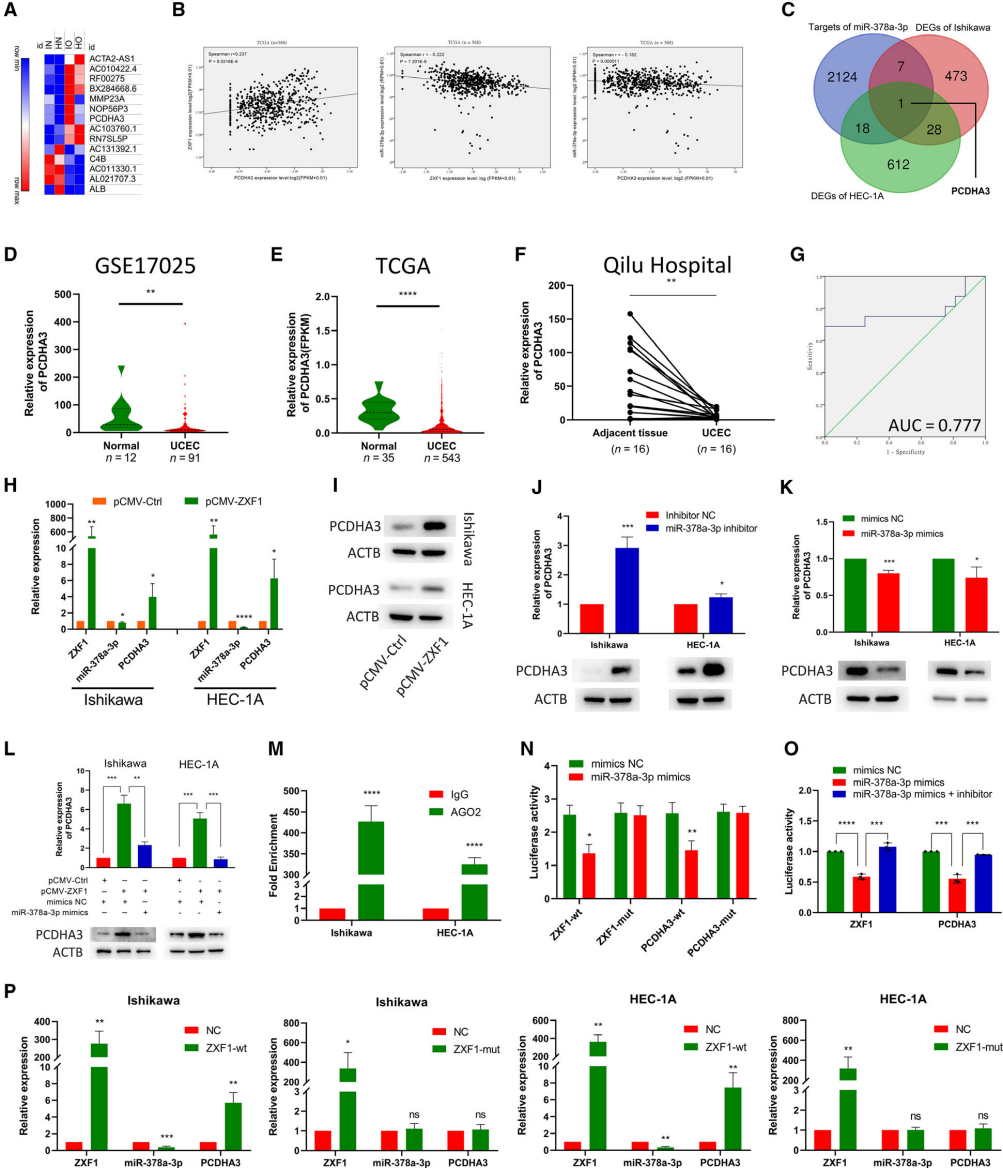
LncRNA‐ZXF1 modulates the miR‐378a‐3p/PCDHA3 axis as a ceRNA. (A) Heatmap of DEGs sequenced after ZXF1 overexpression in endometrial cancer cells. (B) The correlations among ZXF1, miR‐378a‐3p, and PCDHA3 were analyzed using TCGA data. (C) Venn diagram of miR‐378a‐3p target molecules and sequenced DEGs in two cell lines. (D) and (E) PCDHA3 expression in GSE17025 and TCGA data. The GSE17025 (D) and TCGA (E) data revealed lower PCDHA3 expression in cancer tissues than in the control group. The *P*‐value was obtained from the Mann–Whitney U‐test. (F) Among the 16 pairs of clinical samples collected from Qilu Hospital, PCDHA3 expression was higher in tumor adjacent tissues than in tumor tissues. The *P*‐value was obtained from the Mann–Whitney U‐test. (G) The ROC curve of the PCDHA3 mRNA expression level in Qilu Hospital samples indicated that this molecule has potential as a prognostic indicator. (H) After ZXF1 was overexpressed in Ishikawa and HEC‐1A cells, it was detected that miR‐378a‐3p decreased and the PCDHA3 mRNA increased. (I) The PCDHA3 protein raised in EEC cells after ZXF1 overexpression. (J) and (K). The miR‐378a‐3p inhibitor and mimics were transfected into EEC cell lines. The miR‐378a‐3p inhibitor increased the expression of the PCDHA3 mRNA and protein (J), while the mimics produced the opposite result (K). (L) The expression of the PCDHA3 mRNA was restored by miR‐378a‐3p mimics in ZXF1‐overexpressing cells, and the same trend was observed at the protein level. (M) RIP experiments for miR‐378a‐3p were performed in Ishikawa and HEC‐1A cells. MiR‐378a‐3p was more enriched by AGO2 than IgG. (N). Dual‐luciferase assays were used to verify the direct binding of ZXF1 and PCDHA3 to miR‐378a‐3p. Compared with the ZXF1 mutant, miR‐378a‐3p mimics reduced the luciferase activity of wild‐type ZXF1. PCDHA3 produced a similar effect. (O) The luciferase activity of wild‐type ZXF1 was attenuated by miR‐378a‐3p mimics, and this decrease was reversed by the miR‐378a‐3p inhibitor. PCDHA3 produced a similar effect. (P) In the two EEC cells, wild‐type ZXF1 reduced the expression of miR‐378a‐3p and increased PCDHA3, while mutant‐type ZXF1 cannot. All data are mean ± SD. Significance calculated using the unpaired t‐test. *P < 0.05, **P < 0.01, ***P < 0.001,****P < 0.0001. Representative data are from three independent experiments.

We sequenced Ishikawa and HEC‐1A cell lines overexpressing ZXF1 to understand the mechanism by which ZXF1 suppresses cancer growth. Genes with altered expression in the two cell lines were obtained based on a fold change > 2 and *P*‐value < 0.05 as filter conditions. The results of the bidirectional hierarchical clustering analysis of these differentially expressed genes (DEGs) are shown in a heatmap (Fig. 4A). Recently, the most fruitful direction of lncRNA studies is focused on the ceRNA mechanism [19, 20, 21]. We assumed that ZXF1 might also have such a regulatory relationship. We used bioinformatics methods to identify the ceRNA function of ZXF1. In the ceRNA system, lncRNA expression was negatively correlated with miRNA expression but positively correlated with mRNA expression. ENCORI revealed two miRNAs that were negatively correlated with ZXF1 expression, miR‐378a‐3p [22, 23] (Fig. 4B), and miR‐378c. The target gene lists of the two miRNAs were used to make a Venn diagram with the DEG list obtained from the sequencing data. The list of miR‐378a‐3p target genes revealed a common gene, PCDHA3 (Fig. 4C). We assessed the relationship between the expression of PCDHA3 with ZXF1 and miR‐378a‐3p in TCGA database. The expression of ZXF1/miR‐378a‐3p/PCDHA3 supports the ceRNA mechanism (Fig. 4B). Although the TCGA data were statistically significant, the correlation index between these molecules was not very high. Therefore, more experiments are needed to verify the relationship between these molecules.

Based on GEO (Fig. 4D) and TCGA (Fig. 4E) data, the expression of PCDHA3 in tumor tissues was lower than in normal control tissue. qRT‐PCR was performed to verify the change in PCDHA3 expression using samples from Qilu Hospital (Fig. 4F). Additionally, a ROC curve analysis of samples from Qilu Hospital indicated that PCDHA3 can distinguish between adjacent tissues and cancerous tissues with good specificity and sensitivity (Fig. 4G). The same conclusions were drawn from TCGA and GEO data (Fig. S3c). Based on these results, PCDHA3 and ZXF1 have similar expression patterns. In vivo experiments with ZXF1 showed that the expression of miR‐378a‐3p was reduced and the expression of PCDHA3 was increased in tumors overexpressing ZXF1 (Fig. S3d). In EEC cells transfected with ZXF1, miR‐378a‐3p expression was decreased while PCDHA3 expression was increased (Fig. 4H,I). Inhibition of miR‐378a‐3p increased the expression of PCDHA3 (Fig. 4J), and PCDHA3 expression was suppressed after transfection of miR‐378a‐3p mimics (Fig. 4K). As shown in Fig. 4L, the effect of ZXF1 on upregulating PCDHA3 expression was reversed by miR‐378a‐3p. Thus, ZXF1 may function as a miR‐378a‐3p sponge to regulate the expression of PCDHA3. The regulatory effect of miRNA on target genes usually occurs by the interaction of the miRNA with AGO2 to form a complex that degrades the mRNAs of target genes [24, 25]. We conducted RIP and dual‐luciferase reporter experiments to examine whether miR‐378a‐3p regulates the expression of the PCDHA3 mRNA by inducing its degradation. The results of RIP experiments verified that AGO2 bound to miR‐378a‐3p (Fig. 4M). Then, we constructed plasmids carrying the wild‐type and mutant PCDHA3 sequence for dual‐luciferase reporter experiments (Fig. S3e). The luciferase plasmid and miR‐378a‐3p were transfected into HEK293T cells, and fluorescence was measured after 36 h. Notably, miR‐378a‐3p reduced the luciferase activity of wild‐type PCDHA3 and ZXF1, but the luciferase activity of the mutant exhibited no change (Fig. 4N). The miR‐378a‐3p inhibitor rescued the decrease in fluorescence due to miR‐378a‐3p mimics (Fig. 4O). These results indicated that miR‐378a‐3p interacted with AGO2 to degrade PCDHA3. We constructed plasmids carrying the wild‐type and mutant ZXF1 sequences for subsequent experiments designed to explore the role of ZXF1 in this regulatory mechanism. In Ishikawa and HEC‐1A cell lines, wild‐type ZXF1 reduced the expression of miR‐378a‐3p and increased the expression of PCDHA3. However, the expression of miR‐378a‐3p and PCDHA3 did not change significantly after the transfection of mutant ZXF1 (Fig. 4P). Based on these results, ZXF1 could competitively bind to miR‐378a‐3p to protect the mRNA of PCDHA3 from binding and degradation by miR‐378a‐3p.

### 3.5 PCDHA3 functions as a tumor suppressor gene in EEC

**Fig. 5:**
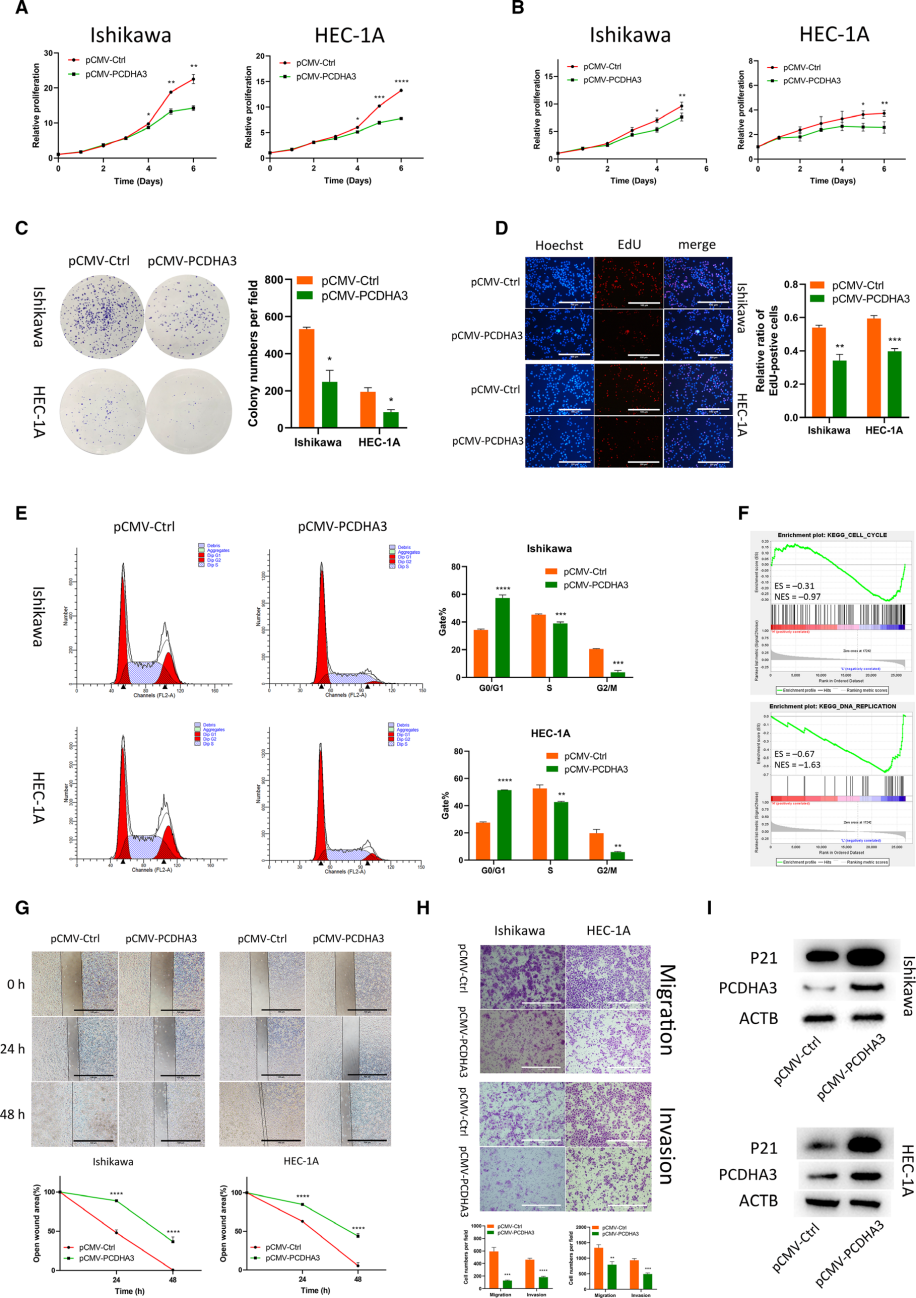
PCDHA3 inhibits the malignant phenotypes of endometrial cancer cells. (A) CellTiter activity was used to detect the proliferation of Ishikawa and HEC‐1A cells. The proliferation of EEC cell lines was inhibited after PCDHA3 overexpression. Comparisons among multiple groups are analyzed using two‐way ANOVA. (B) After pCMV‐PCDHA3 was transfected into endometrial cancer cells, MTT assays proved that PCDHA3 inhibited the proliferation of EEC cells. Comparisons among multiple groups are analyzed using two‐way ANOVA. (C). The amplification of single EEC cells transfected with pCMV‐PCDHA3 in colony formation experiments was suppressed. (D) PCDHA3 overexpression suppressed nuclear DNA replication in Ishikawa and HEC‐1A cells. Scale bars, 100 μm. (E) The cell cycle was detected using flow cytometry. PCDHA3 increased the proportion of EEC cells in G0/G1 phase and decreased the proportion of EEC cells in S and G2/M phase. (F) TCGA data were divided into two groups according to the median expression of PCDHA3. The GSEA using these two datasets showed that cell cycle and DNA replication were enriched. (G) Wound healing experiments were used to explore cell migration capabilities. Detection of the healing rate of the scratches at different time points showed that PCDHA3 prevented the healing of the scratch wounds. Scale bars, 100 μm. (H) The Transwell system was used to detect the migration and invasion capabilities of EEC cells. Compared with the control group, fewer cells transfected with pCMV‐PCDHA3 migrated and invaded. Scale bars, 100 μm. (I) PCDHA3 overexpression increased P21 protein expression in Ishikawa and HEC‐1A cells. All data are mean ± SD. Significance calculated using the unpaired *t*‐test. *P < 0.05, **P < 0.01, ***P < 0.001, ****P < 0.0001. Representative data are from three independent experiments.

The experimental results described above indicate that PCDHA3 may be a tumor suppressor gene. A PCDHA3‐overexpressing lentivirus was transfected into EEC cells to understand its function in EEC and explore its effect on cell proliferation. After PCDHA3 transfection, cell growth was significantly inhibited, as evidenced by the results of the Celltiter‐Glo (Fig. 5A) and MTT (Fig. 5B) assays. The colony formation assay was performed to explore the proliferation of single cells. Although the colony formation capabilities of the two tumor cell lines were different, PCDHA3 overexpression suppressed colony formation in both cell lines (Fig. 5C). We assessed DNA replication in the nucleus by performing EdU staining. Compared with the normal control, PCDHA3‐overexpressing cells exhibited a lower percentage of EdU staining (Fig. 5D). Flow cytometry results showed a higher percentage of PCDHA3‐overexpressing cells in G0/G1 phase and lower percentage of cells in S and G2/M phases (Fig. 5E). TCGA data were divided into two groups according to the expression level of PCDHA3, and a GSEA was performed. Cell cycle and DNA replication were enriched in the results (Fig. 5F). The wound healing (Fig. 5G) and Transwell (Fig. 5H) assays confirmed that PCDHA3 reduced the ability of EEC cells to migrate and invade. The expression of the P21 protein in EEC cells increased upon PCDHA3 overexpression (Fig. 5I). Therefore, PCDHA3 could play a role in EEC as a tumor suppressor gene.

### 3.6 ZXF1 regulates the P21 protein level by modulating ubiquitination

**Fig. 6:**
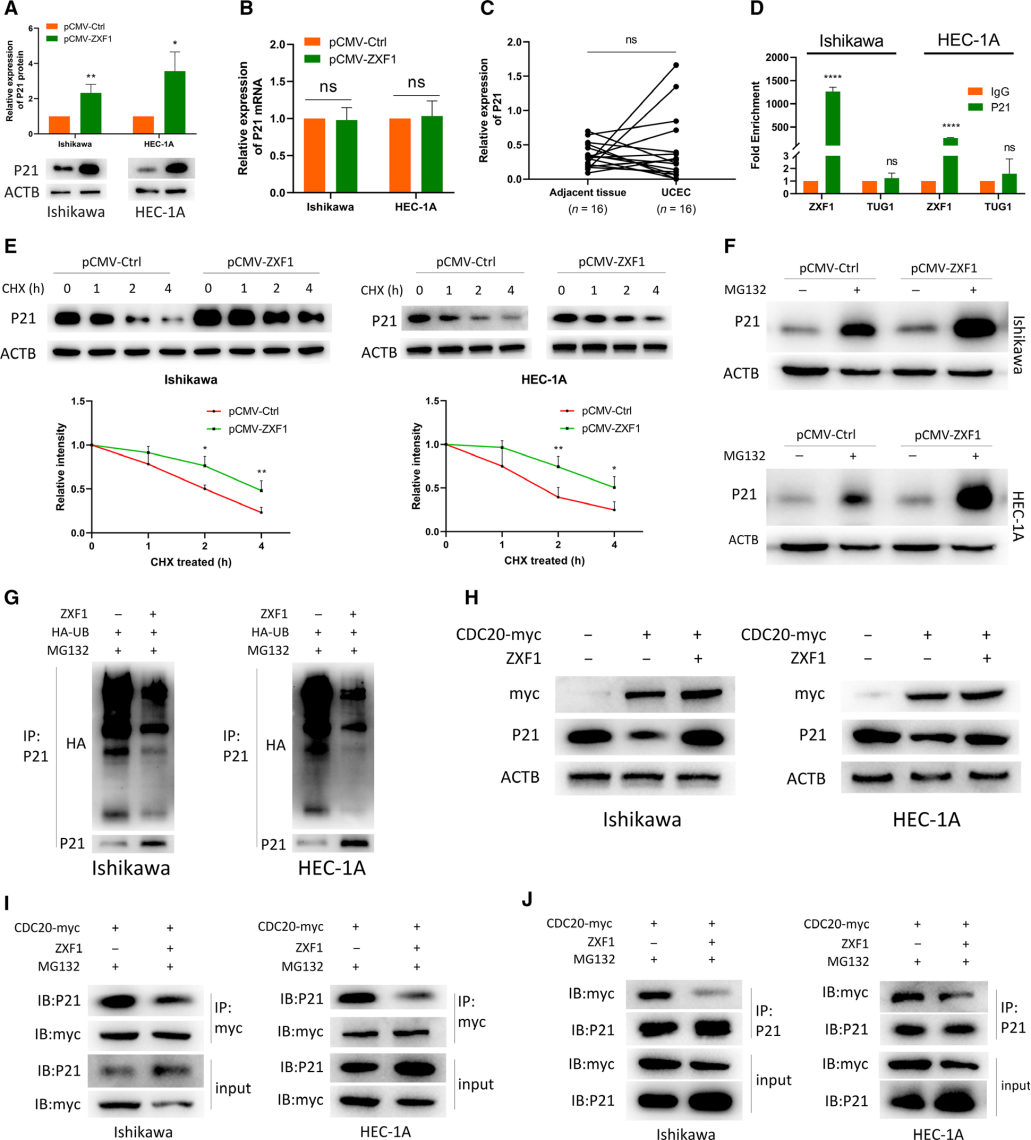
LncRNA‐ZXF1 stabilizes P21 by preventing P21 ubiquitination. (A) ZXF1 overexpression in Ishikawa and HEC‐1A cells increased P21 protein expression. (B) In Ishikawa and HEC‐1A cells overexpressing ZXF1, P21 mRNA expression did not change significantly. (C) No significant difference in the expression of the P21 mRNA was observed between 16 UCEC tissues and adjacent tissues. (D) RIP experiments verified the direct binding of P21 and ZXF1 in Ishikawa and HEC‐1A cells. Compared with IgG, the specific antibody against P21 resulted in a greater amount of ZXF1 bound. LncRNA‐Tug1 served as a negative control. (E) EEC cells were treated with CHX to examine protein degradation. P21 protein degradation was slower in EEC cells transfected with pCMV‐ZXF1 than in the control group. Comparisons among multiple groups are analyzed using two‐way ANOVA. (F) After blocking the function of the proteasome, ZXF1 increased P21 accumulation. (G) The presence of ZXF1 obstructed the ubiquitination of P21. (H) ZXF1 restored the degradation of P21 by CDC20. (I) and (J) ZXF1 prevented the binding of P21 and CDC20 in Ishikawa and HEC‐1A cells. All data are mean ± SD. Significance calculated using the unpaired *t*‐test. *P < 0.05, **P < 0.01, ****P < 0.0001. Representative data are from three independent experiments.

We evaluated the stability of P21 protein expression to determine the role of ZXF1 in cell cycle regulation. After ZXF1 overexpression, P21 levels increased significantly in EEC cell lines (Fig. 6A). However, qRT‐PCR showed no significant changes in the P21 mRNA levels (Fig. 6B). Therefore, ZXF1 may increase the expression of the P21 protein without affecting the expression of the P21 mRNA. We further verified the lack of change in P21 mRNA expression in tumors and adjacent tissues obtained from Qilu Hospital (Fig. 6C). TCGA (Fig. S4a) and GEO (Fig. S4b) data showed the same trend. We conducted a binding experiment using ZXF1 and P21 to determine their relationship. RIP experiments verified the direct binding of ZXF1 to the P21 protein in both Ishikawa and HEC‐1A cell lines (Fig. 6D). Based on these results, ZXF1 regulates the level of the P21 protein, but not its mRNA, through direct binding.

We considered whether the degradation of the P21 protein was regulated by ZXF1. Ubiquitin‐mediated degradation is a common regulated degradation method. EEC cells overexpressing ZXF1 were analyzed at different time points after treatment with cycloheximide (CHX). P21 was degraded more slowly in cells overexpressing ZXF1 and degraded faster in cells in the control group (Fig. 6E). After 2 h of treatment with MG132, the P21 protein accumulated at higher levels in ZXF1‐overexpressing EEC cells (Fig. 6F). Thus, ZXF1 slows the degradation of the P21 protein by the proteasome.

### 3.7 ZXF1 accumulation diminishes the level of ubiquitination on the P21 protein

We explored the changes in the P21 ubiquitination level to confirm the function of ZXF1 in the degradation of P21 protein by the proteasome. A P21 primary antibody was used to precipitate the protein after the HA‐UB vector was transfected into Ishikawa and HEC‐1A cells. All cells were treated with MG132 for 2 h before lysis. Immunoprecipitation revealed that ZXF1 reduced the level of ubiquitinated P21 (Fig. 6G). The GSEA suggested that ZXF1 may inhibit ubiquitination in tumor cells (Fig. S4c). CDC20 is a known ubiquitin E3 ligase for P21 [26, 27]. CDC20 downregulated the expression level of the P21 protein in EEC cells, and ZXF1 rescued this downregulation (Fig. 6H). Immunoprecipitation experiments were performed in two EEC cell lines to further prove that ZXF1 is involved in the CDC20‐mediated regulation of P21. The binding of CDC20 and P21 was reduced after ZXF1 overexpression (Fig. 6I,J). Thus, ZXF1 participates in controlling the ubiquitination of P21 by hindering the binding of CDC20 to P21.

## 4 Discussion

UCEC is one of the most prevalent malignancies worldwide, and its incidence has been increasing in recent years [3]. Therefore, identifying a specific and effective therapeutic target is crucial to personalize and precisely treated UCEC [5]. The cell cycle plays a key regulatory role in the process of immortalization and continuous cell replication in malignant tumors [7, 28]. This feature may be the cytological basis for continued tumor growth and invasion.

As a new research hotspot, accumulating evidence has confirmed that lncRNAs play an important role in biological processes [9]. Recent studies have also suggested roles for lncRNAs in endometrial cancer [10, 29]. In our current study, we found that ZXF1 is differentially expressed in various tumors, including endometrial cancer. Our study is the first to deeply explore the specific role of ZXF1 in EEC and is very important to understand the molecule and inspire future research on ZXF1.

Many publications have confirmed the important role of the cell cycle in endometrial cancer [28, 30]. We conducted proliferation and invasion experiments using endometrial cancer cells to further explore the function of ZXF1. The results of various experiments confirmed that ZXF1 inhibited the proliferation of endometrial cancer cells. Additionally, regulation of the cell cycle was achieved by regulating the level of the P21 protein. In vivo experiments also showed that the growth of subcutaneous tumors in nude mice was suppressed by ZXF1. qRT‐PCR and IHC data supported this conclusion. Bioinformatics analysis of TCGA data also verified the relationship between ZXF1 and the cell cycle in EEC. Thus, ZXF1 is an important molecule involved in regulating the EEC cell cycle. In our study, Transwell and wound healing assays using cells overexpressing ZXF1 proved its function in controlling migration and invasion. Western blotting of EMT markers suggested that ZXF1 may control migration and invasion through the EMT mechanism. Thus, ZXF1 is also involved in regulating proliferation and invasion in EEC.

P21 is a cyclin‐dependent kinase inhibitor that plays a role in regulating the cell cycle [31, 32]. P21 binds to and inhibits the activities of CDK2 and CDK4 complexes in G1 phase [33, 34]. Degradation of P21 releases the inhibitory effect on CDKs, thereby accelerating cell cycle progression [35, 36]. The role of P21 is an important control node in tumorigenesis and development [37, 38, 39, 40]. Degradation of the P21 protein may involve multiple ubiquitin E3 ligases [26, 27]. P21 expression may be regulated in cells by various molecules and pathways [41], and ZXF1 participates in some of these pathways. In the present study, we mainly explored how ZXF1 regulates the P21 protein through two mechanisms.

According to the RNA‐seq analysis and ENCORI prediction, we assumed a regulatory relationship of the ZXF1/miR‐378a‐3p/PCDHA3 axis. In subsequent regulation and rescue experiments, we verified this regulatory pathway. Additionally, PCDHA3 was shown to play a role in the cell cycle and invasion. Based on these results, ZXF1 mediates cell cycle progression and invasion by regulating the expression of PCDHA3. In this process, ZXF1 protects PCDHA3 through the ceRNA mechanism, which has been extensively studied in the context of lncRNAs [19, 20, 21]. This study is the first to explore the function of PCDHA3 in tumors and provides a new perspective for studying malignant tumors. Our results indicate that PCDHA3 modulates P21 protein expression.

The regulation of protein levels through ubiquitin‐mediated degradation is very important for the balance of intracellular P21 [42]. As another mechanism, we found that ZXF1 directly binds to P21 and prevents CDC20‐mediated ubiquitin degradation. CDC20 is an E3 ligase for P21 [26, 43]. Ubiquitin is attached to the P21 protein molecule, which is then recognized by the proteasome and degraded. Our results illustrated that ZXF1 might directly bind to P21 and block the CDC20 binding site, thereby protecting P21 from ubiquitin‐mediated degradation.

The DFS curve also indicated a poor prognosis for patients with low ZXF1 expression. Thus, ZXF1 may help to evaluate EEC disease progression and prognosis. ROC curves from different data platforms indicated that ZXF1 may be a sensitive biomarker of EEC and could predict the diagnosis. These aforementioned findings provide a new direction for the clinical use of ZXF1 as a biomarker to predict the occurrence and development of EEC, as a valuable factor to determine the survival prognosis of patients, and as a sensitive therapeutic target in endometrial cancer.

The lncRNA structure is very unstable and easily degraded by RNase [11]. However, this feature also makes lncRNAs very flexible and able to bind easily to different molecules in cells, such as miRNAs and proteins [10, 44]. Although an increasing number of studies on lncRNAs have been performed, the functions of lncRNAs remain incompletely understood and warrant further investigation.

## 5 Conclusions

**Fig. 7:**
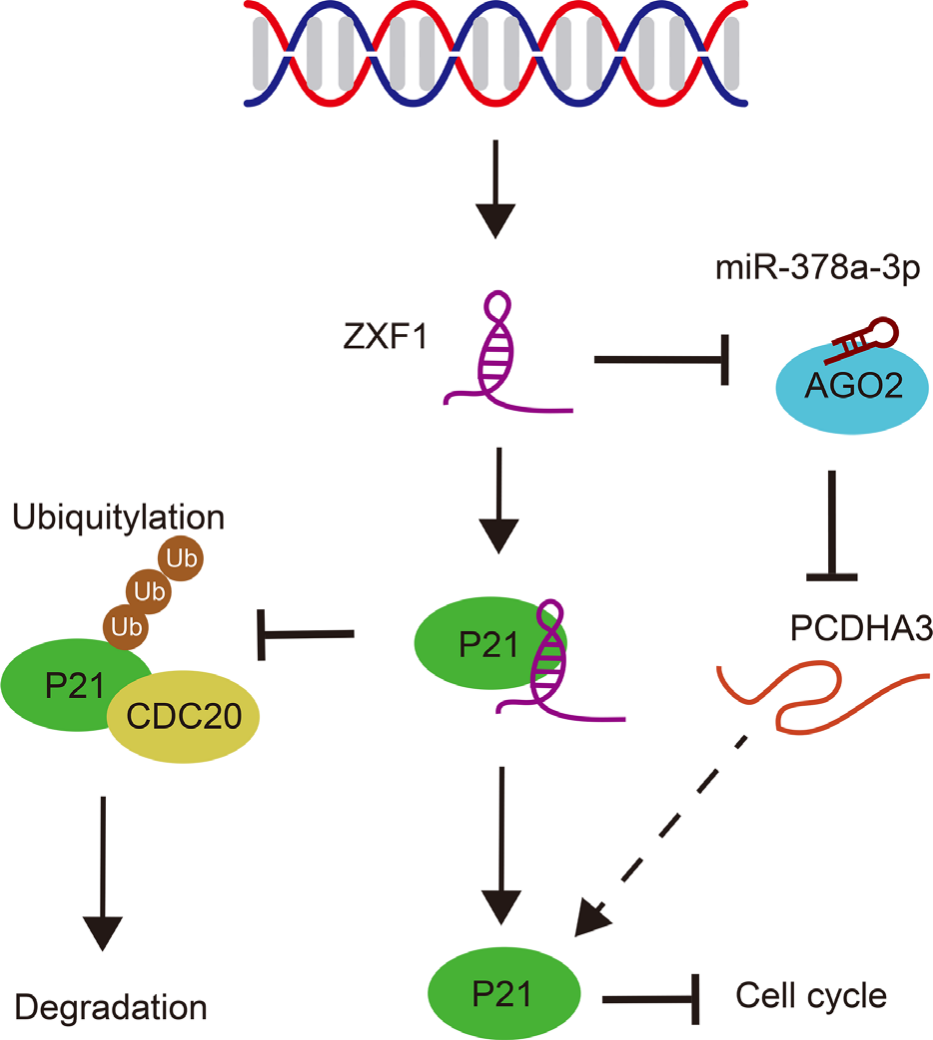
LncRNA‐ZXF1 regulated P21 protein expression and endometrial cancer progression through two mechanisms: inhibiting CDC20‐mediated ubiquitination of P21 by directly binding to P21 and engaging the ZXF1/miR‐378a‐3p/PCDHA3 axis to regulate P21 expression.

In summary, ZXF1 regulates P21 expression in endometrial cancer through two mechanisms: by regulating P21 protein expression through the miR‐378a‐3p/PCDHA3 axis and by directly binding to P21 to prevent its CDC20‐mediated ubiquitin degradation (Fig. 7). Additionally, increased expression of ZXF1 inhibits cell cycle progression and the invasion of endometrial cancer cells. Based on our results, ZXF1 has the potential to become a diagnostic indicator, prognostic indicator, and target for molecular targeted treatment in precision therapy. To the best of our knowledge, this study is the first to show that lncRNA‐ZXF1 functions as a tumor suppressor lncRNA in EEC.

## Declarations

### Ethics approval and consent to participate

All patients were informed by the experimental details and signed informed consent. The experimental procedure complied with the Helsinki Declaration and was authorized by Ethic Committee on Scientific Research of Shandong University Qilu Hospital. All animal experiments were reviewed and approved by Laboratory Animal Ethical and Welfare Committee of Shandong University Cheeloo College of Medicine.

## Author Contributions

JJ conceived and supervised the project. DK conducted all basic medical experiments and collected data. DK, WL, YH, and XM analyzed the data. DK wrote the manuscript. All authors reviewed the manuscript and consent for publication.

## Conflict of interests

The authors declare no conflict of interest.

## Availability of data and materials

All data generated or analyzed during this study are included in this published article [and its supplementary information files].

## Supporting information


**Figure S1.** The expression of ZXF1 in a variety of malignant tumors.
**Figure S2.** The Characteristics of ZXF1 in UCEC.
**Figure S3.** The transfection efficiency of miR‐378a‐3p and PCDHA3, ROC analysis of PCDHA3.
**Figure S4.** RNA expression of P21 and GSEA analysis of ZXF1.
**Table S1.** Antibodies and drugs.
**Table S2.** Primers and nucleotide sequence.
