# Supplementary material for: LncRNA‐ZXF1 stabilizes P21 expression in endometrioid endometrial carcinoma by inhibiting ubiquitination‐mediated degradation and regulating the miR‐378a‐3p/PCDHA3 axis
Source: Mol Oncol. 2021 Mar 22;16(3):813–29. doi: 10.1002/1878-0261.12940 (PMC8807357; doi:10.1002/1878-0261.12940)
Supplement: Supplementary file 1 — Figure S1. The expression of ZXF1 in a variety of malignant tumors. Figure S2. The Characteristics of ZXF1 in UCEC. Figure S3. The transfection efficiency of miR‐378a‐3p and PCDHA3, ROC analysis of PCDHA3. Figure S4. RNA expression of P21 and GSEA analysis of ZXF1. Table S1. Antibodies and drugs. Table S2. Primers and nucleotide sequence. [file MOL2-16-813-s001.pdf]

S1

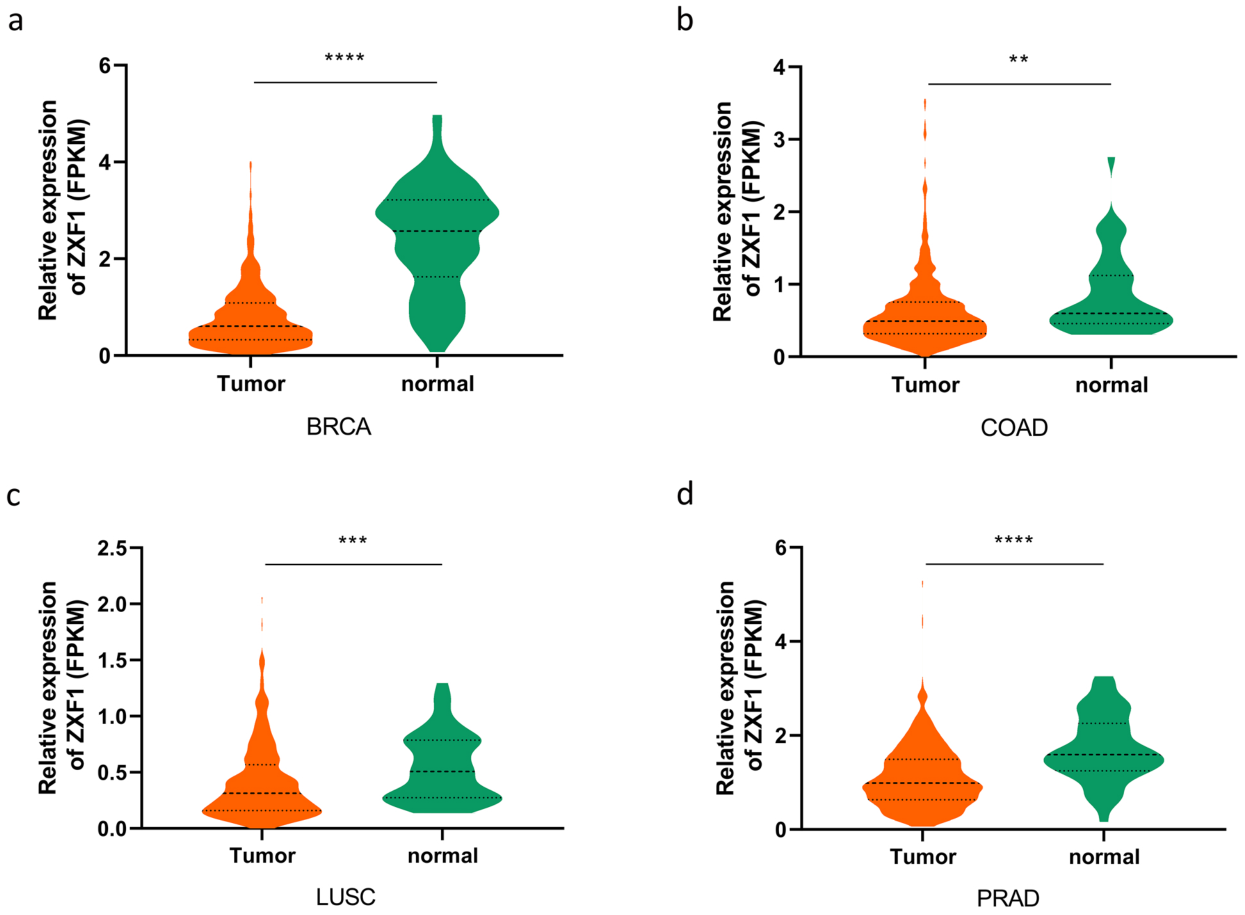

S1 ZXF1 expression in several tumors of TCGA. In BRCA, COAD, LUSC and PRAD, the expression of ZXF1 was lower in tumor tissues than in normal controls. **P＜0.01, ***P＜0.001, ****P＜0.0001 by Mann-Whitney test.

S2

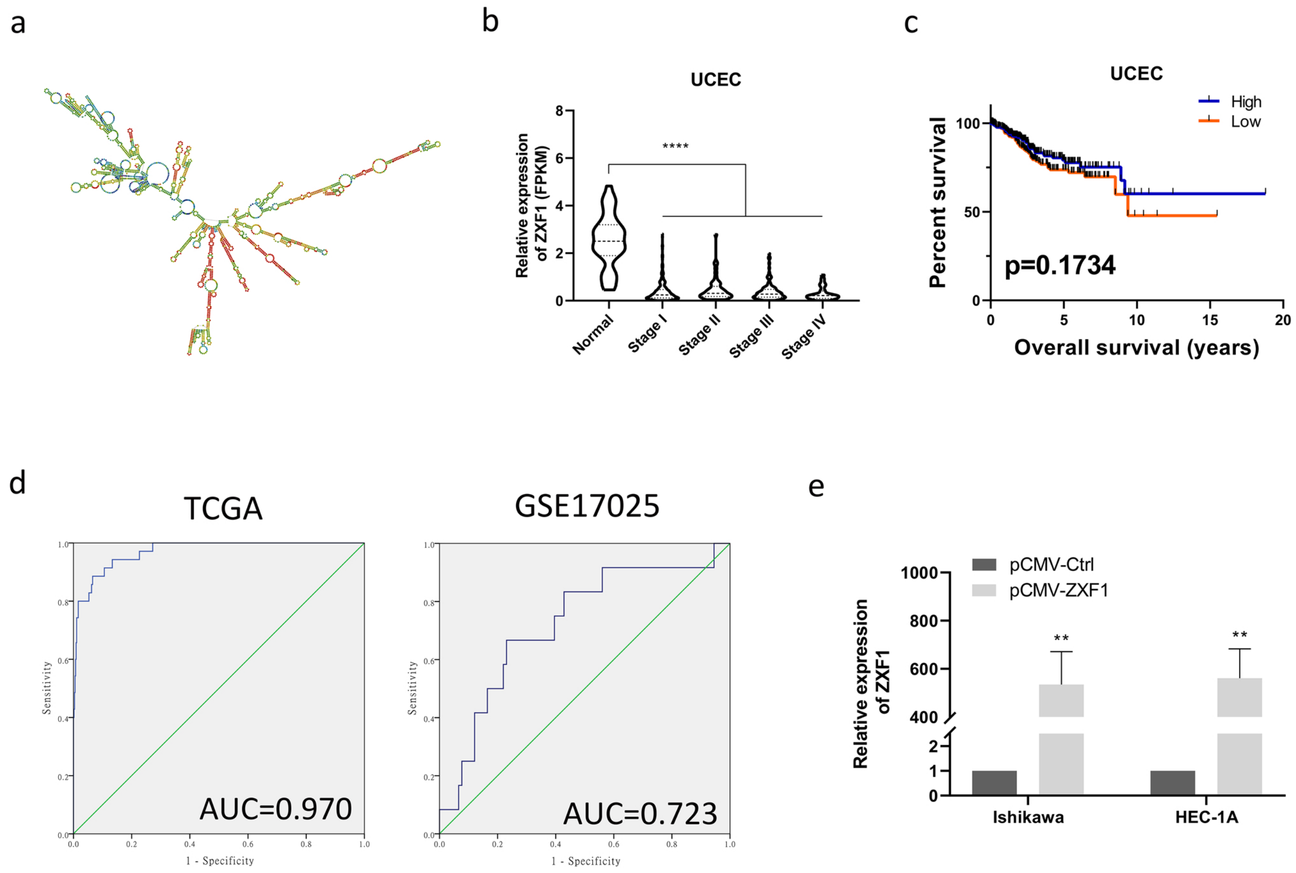

S2 a. Predicted secondary structure of ZXF1 (http://rna.tbi.univie.ac.at). b. Different expressions of UCEC at various stages. In the TCGA data, ZXF1 was statistically different between the normal control group and each tumor stage. But there was no difference between different tumor stages. c. Overall survival(OS) was analyzed using TCGA survival data, with the median as the boundary to divide the samples into two groups. d. ROC analysis of ZXF1 was performed using expression data from TCGA and GSE17025, respectively. e. After overexpression of ZXF1 in Ishikawa and HEC-1A, qRT-PCR was used to detect the overexpression efficiency.

S3

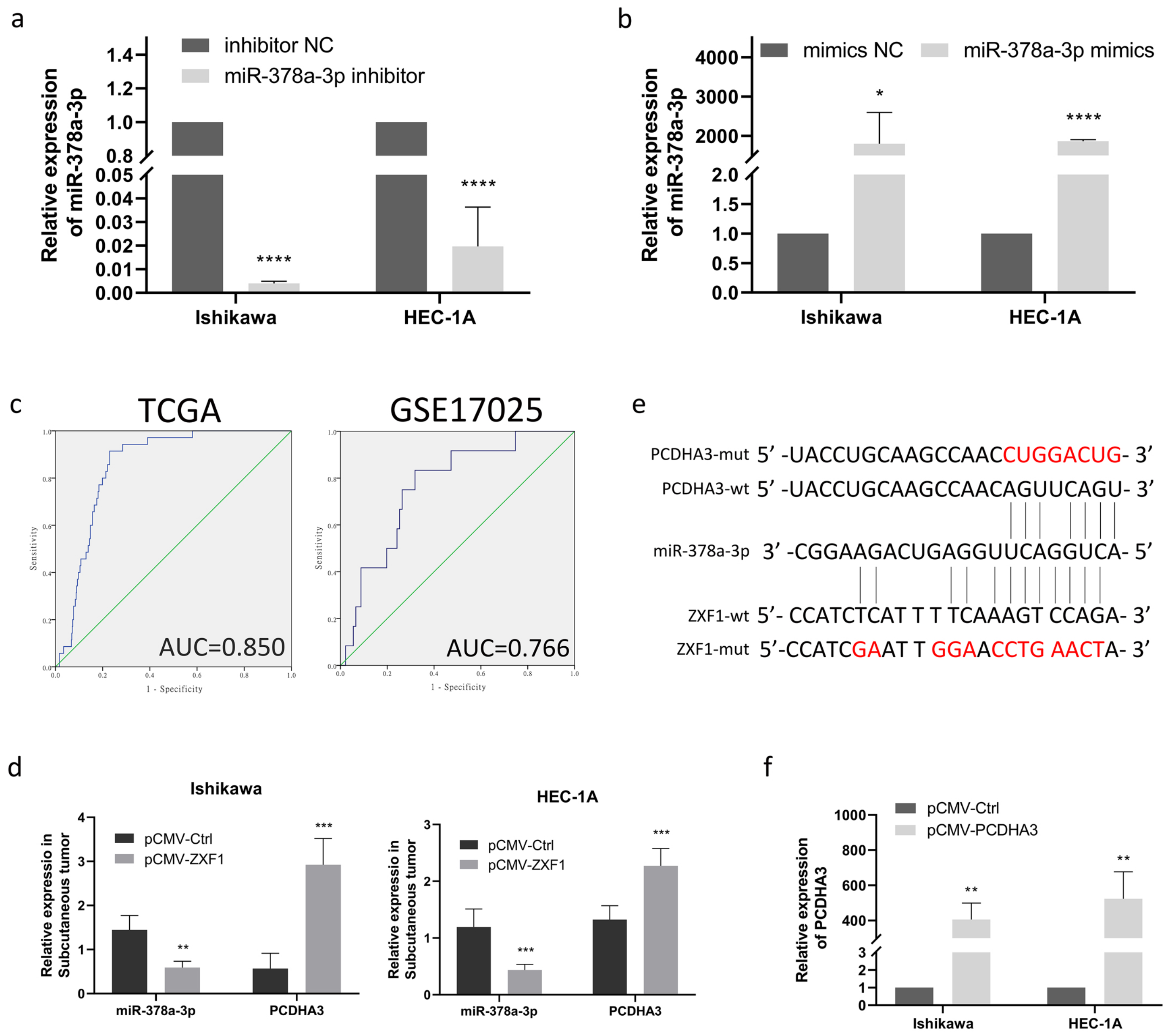

S3 a-b. The transfection efficiency was measured after transfection of miR-378a-3p inhibitor and mimics in EEC cell lines. c. Analysis of PCDHA3 expression data in TCGA and GSE17025, ROC analysis was consistent with the results of Qilu Hospital. The ROC curve shows that PCDHA3 has good sensitivity and specificity as a diagnostic marker. d. In tumors obtained in in vivo experiments, the expression of miR-378a-3p decreased, while PCDHA3 increased. e. The sequence of wild type and mutant type of PCDHA3 and ZXF1 constructed in the dual luciferase report experiment. f. PCDHA3 was transfected into Ishikawa and HEC-1A cells and used to detect transfection overexpression efficiency.

S4

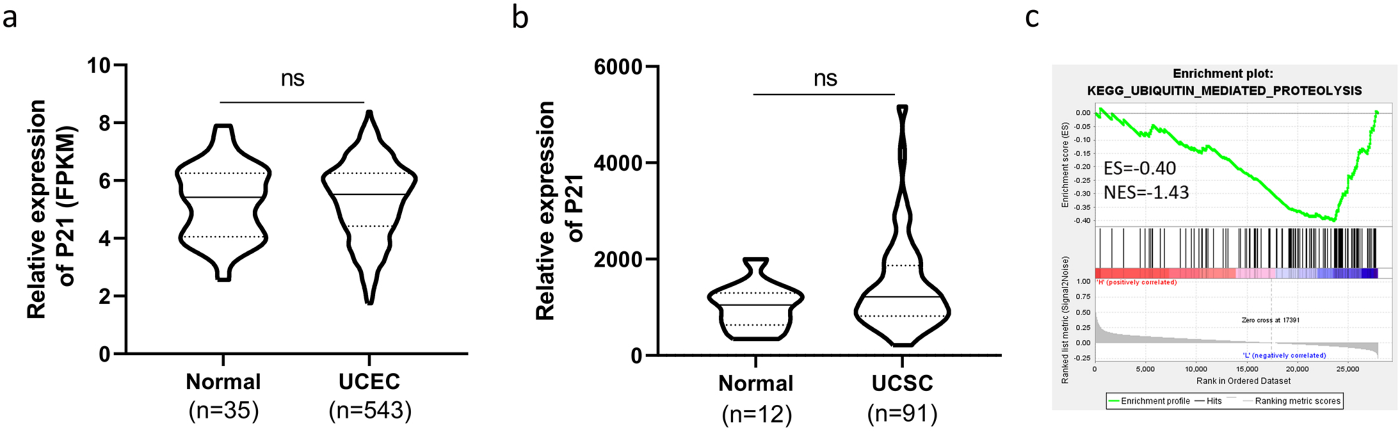

S4 a-b. In UCEC data of TCGA (a) and GSE17025 (b), there was no difference in the expression of P21 between tumor tissue and normal control group. c. All samples were divided into two groups according to the expression of ZXF1 in TCGA, and then GSEA analysis was performed. The result showed that Ubiquitin mediated proteolysis was enriched.

Supplementary Table. 1 Antibodies and drugs used in this experiment

Antibody/Chemicals	Company	Catalog#	Species	Experiment
ACTB	CST	4970	Rabbit	Western blot(1/1000)
P21	CST	2947	Rabbit	Western blot(1/1000),IHC(1/50), RIP(1/50),co-IP(1/50)
MMP2	Abcam	ab92536	Rabbit	Western blot(1/1000)
E-cadherin	CST	14472	Mouse	Western blot(1/1000)
N-cadherin	CST	13116	Rabbit	Western blot(1/1000)
CDC20	Proteintech	10252-1-AP	Rabbit	Western blot(1/1000),co-IP(1/50)
PCDHA3	Proteintech	18803-1-AP	Rabbit	Western blot(1/1000),co-IP(1/50)
Flag-tag	CST	14793	Rabbit	Western blot(1/1000),co-IP(1/50)
AGO2	CST	2897	Rabbit	RIP(1/50)
Myc-tag	Proteintech	16286-1-AP	Rabbit	Western blot(1/1000),co-IP(1/50)
HA	Affinity	T0050	Rabbit	Western blot(1/1000)
Ki67	Servicebio	GB13030-2	Rabbit	IHC(1/200)
Cycloheximide	CST	2112		
MG132	Selleckchem	S2619		
D-Luciferin, Potassium Salt D	YEASEN	40902ES02		

Supplementary Table. 2 Primers and nucleotide sequence used in this experiment

Gene Symbol	Forword primer	Reverse primer
ZXF1	CTACCGATGAAGGATGGCTGG	ACCTGTGCAGACCCTAATGTT
ACTB	CATCCCCCAAAGTTCACAAT	AGTGGGGTGGCTTTTAGGAT
PCDHA3	CTCACTGGCACGACTCAAC	GGCTTCTACCTGGATTTCATA
P21	GACCTGTCACTGTCTTGTACCC	AAGATCAGCCGGCGTTTG
CDC20	TTCCTTCCCTGCCAGACCGTA	TGCCCACAGCCAAGTAGTT
U6	CAGCACATATACTAAAATTGGAACG	CGAATTTGCGTGTCATCC
miR-378a-3p	ATGGTGGACTGGACTTGGAGT	GTGCAGGGTCCGAGGT
miR-378a-3p inhibitor	GCCUUCUGACUCCAAGUCCAGU	
miR-378a-3p mimics	ACUGGACUUGGAGUCAGAAGGC CUUCUGACUCCAAGUCCAGUUU	
